# A proteome-wide immuno-mass spectrometric identification of serum autoantibodies

**DOI:** 10.1186/s12014-019-9246-0

**Published:** 2019-06-20

**Authors:** Milena Music, Antoninus Soosaipillai, Ihor Batruch, Ioannis Prassas, Dimitrios P. Bogdanos, Eleftherios P. Diamandis

**Affiliations:** 1 Department of Laboratory Medicine and Pathobiology, University of Toronto, Toronto, Canada; 2 Department of Pathology and Laboratory Medicine, Mount Sinai Hospital, 60 Murray St [Box 32]; Flr 6 - Rm L6-201-1, Toronto, ON M5T 3L9 Canada; 3 Department of Rheumatology and Clinical Immunology, Faculty of Medicine, School of Health Sciences, University of Thessaly, Biopolis, 41110 Larissa, Greece; 4 Department of Clinical Biochemistry, University Health Network, Toronto, Canada; 5 Lunenfeld-Tanenbaum Research Institute, Mount Sinai Hospital, 60 Murray St [Box 32]; Flr 6 - Rm L6-201-1, Toronto, ON M5T 3L9 Canada

**Keywords:** Immuno-MS, Autoantibodies, Immunoprecipitation, Protein G magnetic beads, Mass spectrometry, Biomarkers, Proteomics

## Abstract

**Background:**

Autoantibodies are produced when tolerance to self-antigens is broken and they can be mediators of tissue injury and systemic inflammation. They are excellent biomarkers because they are minimally invasive to screen and are highly abundant in serum due to limited proteolysis and slow clearance. Conventionally used methods of identifying autoantibodies in patient sera include indirect immunofluorescence, enzyme-linked immunoabsorbent assays (ELISAs) and protein microarrays. Here we present a novel proteome-wide immuno-mass spectrometric method to identify serum autoantibody targets.

**Methods:**

Serum samples from patients with inflammatory bowel disease (IBD) were analyzed by ELISA for the presence of autoantibodies to CUB and zona pellucida-like domain-containing protein 1 (CUZD1). Protein was extracted from the human pancreas as well as 16 other human tissues to make a complex tissue lysate protein mixture. Antibodies in patient sera were immobilized and purified on protein G magnetic beads and subsequently incubated with pancreatic lysate containing CUZD1 or the aforementioned complex tissue lysate. After extensive washing, antibody-bound protein antigens were trypsin-digested and identified using shotgun mass spectrometry.

**Results:**

The protocol was optimized for the immunoaffinity purification of autoantibody targets from tissue lysate, using CUZD1 from pancreatic lysate and anti-CUZD1 autoantibodies present in IBD patient serum as a proof-of-concept. Pancreatic secretory granule membrane major glycoprotein 2, whose autoantibodies are a known biomarker of Crohn’s disease, was also immunoprecipitated from IBD patient serum, as an additional internal positive control.

**Conclusions:**

This study demonstrates the effectiveness of a proteomic approach to identify serum autoantibody targets, using immunoaffinity purification followed by tandem mass spectrometry. Our methodology is applicable for proteome-wide analysis of autoantibody targets in a wide variety of clinical settings.

**Electronic supplementary material:**

The online version of this article (10.1186/s12014-019-9246-0) contains supplementary material, which is available to authorized users.

## Introduction

Antibodies that recognize and react with an individual’s own antigens, called self-antigens, are referred to as autoantibodies [1]. The production of autoantibodies is usually elicited as a result of a breach of tolerance against self-antigens, or due to the modification of self-antigens [2]. Self-antigens comprise proteins, carbohydrates, nucleic acids, lipids or combinations of these antigen types [1]. Autoantibodies play a major role in disease pathogenesis as mediators of both tissue injury and systemic inflammation [2]. They can serve as specific biomarkers for the early diagnosis of some autoimmune diseases, as they may be present before disease onset and clinical manifestation, allowing for appropriate therapeutic interventions [1]. The sera of cancer patients commonly contain several autoantibodies that may be produced due to inflammation, loss of immune tolerance, changes in protein expression levels, mutations and other alterations of protein structure that frequently occur during cancer [3]. Autoantibodies targeting specific tumor-associated antigens are also highly sought after as potential diagnostic and prognostic cancer biomarkers [4]. Additionally, autoantibodies are currently being investigated as predictive biomarkers for immune-related adverse events (irAEs) that manifest as a result of anti-neoplastic treatment with immune checkpoint blockade [5, 6]. Screening for autoantibodies is non-invasive and simple, as they are found in the sera of patients [4]. An advantage of autoantibodies as biomarkers is that they are produced in large amounts in serum, despite low quantities of the corresponding antigen [7]. Furthermore, autoantibodies undergo limited proteolysis and clearance, and have a half-life that is longer than 7 days [4].

**Fig. 1:**
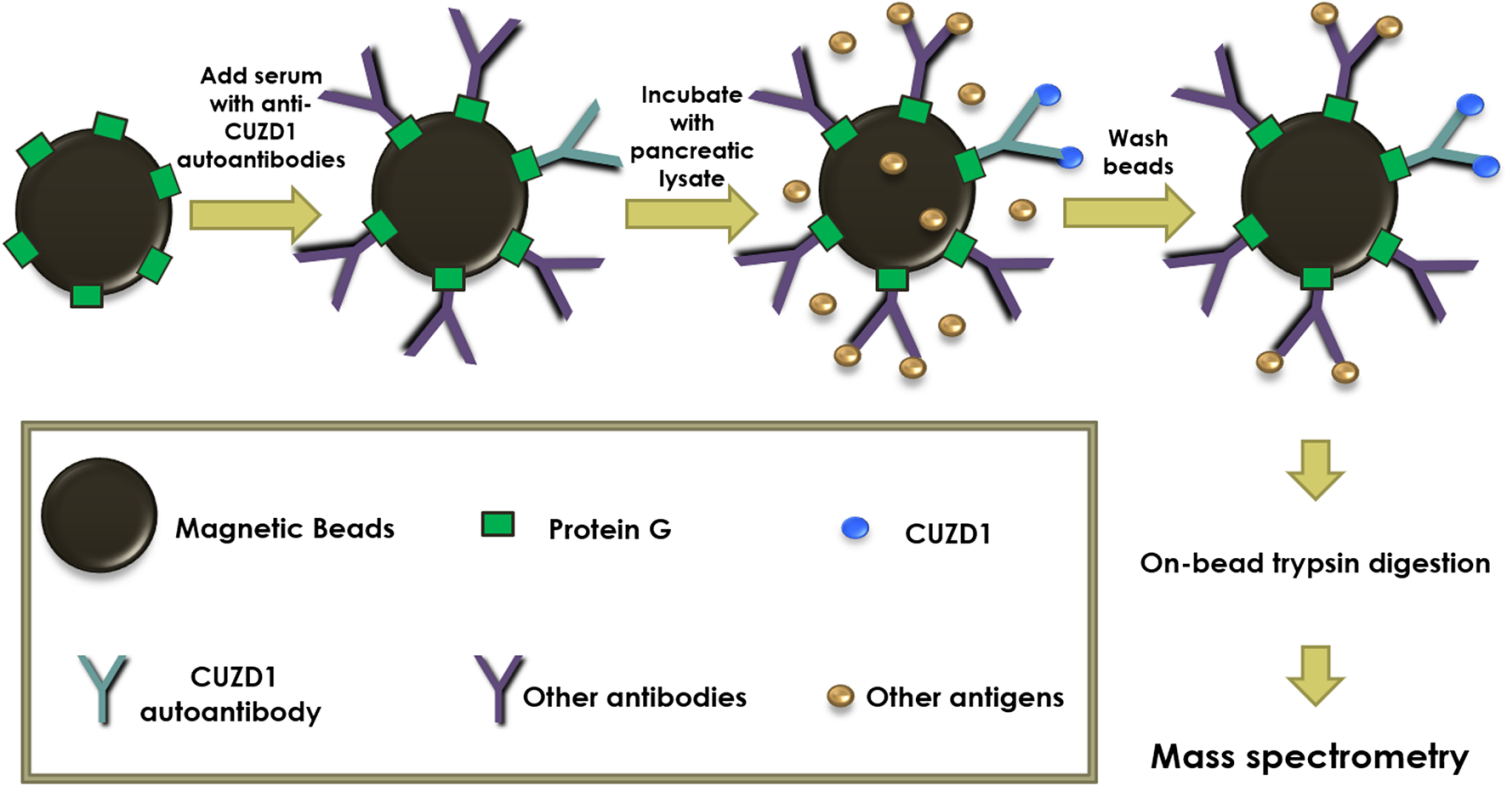
Methodology used to identify anti-CUZD1 serum autoantibodies with protein G magnetic beads. In the first step, serum is added to the beads, to capture serum antibodies, some of which are anti-CUZD1 autoantibodies (blue color). After washing, the beads are exposed to a protein mix, in this case pancreatic lysate or a more complex tissue lysate. In the third step, beads are washed and the captured proteins (including CUZD1; blue dots) are trypsin-digested before identification with tandem mass spectrometry. For more details see text

Currently established methods to identify antigen-specific autoantibodies in patient sera include indirect immunofluorescence, enzyme-linked immunoabsorbent assays (ELISAs), protein microarrays, phage display methods, multiple affinity protein profiling (MAPPing), serological analysis of recombinant tumor cDNA expression libraries (SEREX) and serological proteome analysis (SERPA) [7]. We here present an efficient immuno-mass spectrometry (immuno-MS) approach for the identification of serum autoantibodies. First, all the IgG antibodies, including the autoantibodies, in serum are captured and purified on protein G beads, followed by addition of a protein mix containing thousands of human proteins. This is followed by exhaustive washing of the beads, captured antigen digestion by trypsin and shotgun tandem mass spectrometry (MS) analysis (Fig. 1). Protein G is a streptococcal bacteria cell wall protein that has a very high affinity for immunoglobulin G (IgG), and can be used to purify IgG from the sera of various species [8]. MS is a powerful tool for the precise identification and quantification of the constituents in a complex protein sample [9].

In order to demonstrate that our proposed new method of identifying autoantibodies in serum works effectively, we performed several proof-of-concept experiments involving sera with natural autoantibodies against CUB and zona pellucida-like domain-containing protein 1 (CUZD1), as identified by ELISA and immunofluorescence. CUZD1 is a highly pancreas-specific protein that is distinctly conserved among species [10]. Detectable pancreatic autoantibodies can be found in the sera of up to 30% of patients with Crohn’s disease (CD) [11]. CUZD1 is specifically linked to inflammatory bowel disease (IBD), as autoantibodies elicited against it have been proposed as a novel serological biomarker of CD [12]. Here, we show that our immuno-MS approach is an effective strategy for identifying proteome-wide autoantibody targets in patient sera.

## Methods

### Sample collection

The Institutional Review Boards of Mount Sinai Hospital, University Health Network and the University of Thessaly approved all of our protocols, including collection of tissue and serum samples. Tissues were obtained from Mount Sinai Hospital and the University Health Network at autopsy. Serum samples from patients with IBD, which were previously assigned as positive or negative for anti-CUZD1 autoantibodies by immunofluorescence, were obtained from the University of Thessaly.

### Tissue protein extraction

Tissues were stored at − 80 °C until use. Protein was extracted from 17 tissues: lung, esophagus, gallbladder, heart, adipose tissue, small intestine, skeletal muscle, endometrium, prostate, breast, spleen, bone marrow, bladder, duodenum, stomach, kidney and pancreas. For protein extraction, tissue was pulverized in liquid nitrogen with a mortar and pestle to yield a fine powder. 0.1–0.2% RapiGest SF Surfacant (Waters, Milford, MA, USA) in 50 mM ammonium bicarbonate (ABC) was added to further lyse the tissues. The pulverized tissue sample was vortexed every 5–10 min on ice, for 30 min, and then sonicated on ice for 15 s, three times, to further disrupt the cells. Following sonication, the sample was centrifuged at 15,000 g for 20 min at 4 °C, to remove debris and insoluble contents, followed by collection of the supernatant. A Pierce BCA Protein Assay (Thermo Fisher Scientific, San Jose, California) was performed on tissue lysate protein extracts for total protein quantification.

### ELISA

**Fig. 2:**
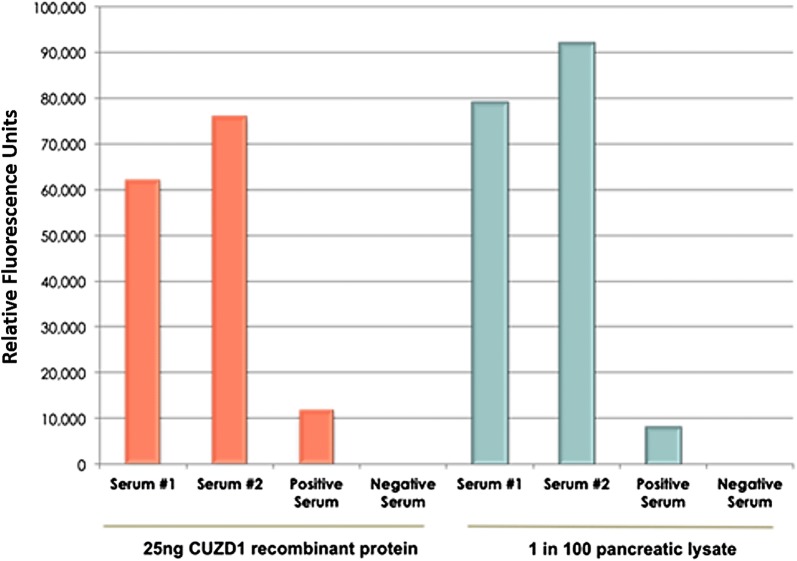
ELISA assay for identifying anti-CUZD1 serum autoantibodies. The method is described in detail in reference 12. Two different sera (Serum #1 and Serum #2) were tested against a previously confirmed positive serum control as well as a negative serum control. The fluorescence signal from the blanks was removed from each result. The two sera had higher autoantibody titers than the previously identified positive control. These sera were used to develop our MS-based assay

A sandwich-type ELISA was performed to confirm the presence of anti-CUZD1 serum autoantibodies in two IBD patient samples obtained from the University of Thessaly. White polystyrene microtiter plates were coated overnight at room temperature with 100 µl of anti-CUZD1 primary mouse antibody in coating buffer (50 mM TRIS base and 0.05% NaN_3_, pH 7.8) at a final concentration of 5 ng/µl. The next day, the plate was washed three times with washing buffer (5 mM TRIS base, 150 mM NaCl, 0.05% Tween 20, pH 7.8) using an automatic washer and incubated with 100 µl/well of CUZD1 protein (see below) for 2 h with shaking. Wells were incubated with either 25 ng of recombinant CUZD1 protein in 6% bovine serum albumin (BSA) or with pancreatic lysate (extracted from human pancreas) diluted 100-fold in 6% BSA. After incubation, the plate was washed three times and incubated for 1 h shaking, with 100 µl/well of serum samples (Fig. 2), diluted 1:500 in green assay buffer (60 g/L BSA, 0.1 g/L goat globulin, 0.02 g/L mouse globulin, 1 g/L bovine globulin, 5 ml/L Tween 20 and 37 g/L KCl). Positive and negative control sera were previously confirmed for their presence or absence of anti-CUZD1 autoantibodies by ELISA [12]. Following three washes, the plate was incubated for 1 h with 100 µl/well of alkaline phosphatase-conjugated goat anti-human secondary antibody (H + L specific) diluted 10,000 fold in green assay buffer. The plate was then washed six times and incubated for 10 min with diflunisal phosphate solution (0.1 M NaOH containing 10 mM diflunisal phosphate) diluted 1 in 20 in substrate buffer (0.1 M Tris, 0.15 M NaCl, 1 mM MgCl_2_ and 0.05% NaN_3_, pH 9.1). The reaction was stopped with 100 µl/well of developing solution (1 M Tris, 0.15 M NaOH, 2 mM TbCl_3_ and 3 mM EDTA). Fluorescence at 450 nm was measured with a time-resolved fluorometer, EnVision (Perkin-Elmer).

### Immunoprecipitation on protein G magnetic beads

Depending on the experimental set-up, a minimum of 100 µl of 10% w/v Protein G Mag Sepharose Xtra magnetic beads (GE Healthcare) medium slurry was resuspended by vortexing and added to a centrifuge tube (Additional file 1: Table S1). The beads have a capacity of > 27 µl human IgG/µl magnetic beads. The tube was added to a magnetic separator for centrifuge tubes, to remove the storage solution from the beads. The magnetic beads were equilibrated with 500 µl of phosphate buffered saline (PBS) for 100 µl of beads slurry, followed by removal of PBS through use of the magnet. Serum that is highly positive for anti-CUZD1 autoantibodies (Serum #2) was diluted 12 to 120-fold to a final volume of 300 µl in PBS and added to protein G magnetic beads. IgG in the serum was bound to the beads during a 30–60 min incubation with gentle rotation. After two washes with at least 500 µl PBS, the beads were incubated with at least 20 µg of total protein from pancreatic lysate or 850 µg of a complex tissue lysate mixture for 2 h using gentle rotation. During this time, the autoantibodies bound to their cognate proteins in the protein mixture. Following incubation, three washes with at least 500 µl PBS 0.05% Tween 20 were performed to remove unbound proteins. An additional three washes with at least 500 µl PBS were performed to remove Tween 20 detergent (this detergent is not compatible with MS). The beads were resuspended in at least 100–200 µl of PBS for on-bead trypsin digestion.

### On-bead protein digestion for mass spectrometry

The immuno-captured autoantibodies and target antigens on protein G magnetic beads were reduced with 100 mM dithiothreitol (DTT) added to a final concentration of 5 mM and incubated for 40 min at 56 °C. The samples were then alkylated with 500 mM iodoacetamide (IAM) added at a final concentration of 15 mM and incubated for 30 min at room temperature, in the dark and with shaking. Trypsin was added in a 1:50 ratio (protease to total beads IgG capacity) and the samples were incubated overnight at 37 °C with shaking. The following morning, the immunoprecipitated target antigens were separated from the protein G magnetic beads using a magnet. Formic acid was added to a final concentration of ~ 1% to acidify the samples to pH 2 and inactivate trypsin activity.

### Analysis of immunoprecipitated samples by mass spectrometry

In all samples, peptides were extracted from solution using C18 OMIX tips (Agilent Technologies, Santa Clara, CA) and eluted in 3 µl of elution buffer B (65% acetonitrile, 0.1% formic acid). Fifty-seven μl of buffer A (0.1% formic acid) was added to each sample and 18 μl of sample was loaded from a 96-well microplate autosampler onto a C_18_ Acclaim PepMap 100 (75 μm × 2 cm, C18 3 μm bead, 100 Å pore size) trap column (Thermo Fisher Scientific, San Jose, California) using the EASY-nLC 1200 system (Thermo Fisher Scientific) and running buffer A (0.1% formic acid). Peptides were eluted from the trap column at 250 nl/min with an increasing concentration of Buffer D (0.1% formic acid in 95% acetonitrile) over a 60-min gradient onto a resolving 50 cm long analytical column (PepMap RSLC C18, 75 μm ID, 2 μm bead, 100 Å pore, ES803, Thermo Fisher Scientific). The liquid chromatography setup was coupled online to Q Exactive HF-X (Thermo Fisher Scientific) mass spectrometer using the EASY-Spray ionization source (Thermo Fisher Scientific) with the capillary temperature set to 320 °C and a spray voltage of 2 kV. A 60-min data-dependent acquisition method performed a full MS [1]  scan from 400 to 1500 *m*/*z* at a resolution of 60,000 in profile mode. This was followed by fragmentation of the top 28 parent ions using the HCD cell and detection of fragment ions at a resolution of 15,000. The following MS method parameters were used: MS1 Automatic Gain Control target was set to 3e6 with maximum injection time (IT) of 100 ms; MS2 AGC was set to 1e5 with maximum IT of 22 ms; isolation window was 1.6 m/z; intensity threshold was 4.5e4; normalized collision energy was set to 27; charge exclusion was set to fragment only 2 +, 3  +, 4 + and 5 + charge state ions; peptide match was set to preferred and dynamic exclusion was set to 20 s.

### Bioinformatics

All mass spectrometry RAW files were uploaded into Proteome Discoverer v. 1.4 (Thermo Fisher Scientific) and searched with the Human 5640 Swiss-Prot protein database (January 2018). The following parameters were used for the search: Sequest HT search engine; trypsin (full) enzyme with up to 2 missed cleavages allowed; precursor mass tolerance of 7 ppm and fragment mass tolerance of 0.02 Da; carbamidomethylation of cysteine was set to static modification; oxidation of methionine was set to dynamic modification; peptide and protein level false-discovery rate was set at 1% using the Percolator node.

## Results

### Tissue lysate protein concentrations

We prepared lysates from 17 human tissues and extracted the proteins as described. The total protein content of each lysate ranged from 1.1 to 13 mg/ml (Additional file 2: Table S2).

### Identification of anti-CUZD1 autoantibodies by ELISA

We tested by in-house ELISA a previously identified positive serum for CUZD1 autoantibodies (positive serum), one negative serum and an additional two IBD patient sera (Serum #1 and Serum #2) which were highly positive for CUZD1 autoantibodies by immunofluorescence. The ELISA confirmed that both tested IBD patient sera were highly positive for anti-CUZD1 autoantibodies and had a high ELISA fluorescence signal in comparison to the negative control serum (Fig. 2). The two tested IBD samples showed over fivefold higher fluorescence than the previously confirmed positive control serum.

### Immuno-mass spectrometric assay optimization

We used the protocol described in Fig. 1 to identify autoantibody targets in the tested sera. The critical parameters of the assay were volume of serum, amount of protein lysate and amount of protein G magnetic beads. Additional file 1: Table S1 summarizes the combinations of these parameters. We found that optimal results (positive identification of CUZD1 as an antibody target) were obtained when the serum volume was varied between 2.5 and 25 µl and the amount of pancreatic tissue lysate total protein concentration ranged from 20 to 100 µg. We also found that 100 µl of 10% w/v magnetic beads slurry was sufficient to bind a representative amount of IgG in 25 µl of serum. Additionally, we optimized carefully the amount of washes (3 × with PBS 0.05% Tween 20 and 3 × with PBS only) so that non-specific absorption of proteins on the beads is minimized.

### Identification of CUZD1 and GP2 proteins

**Fig. 3:**
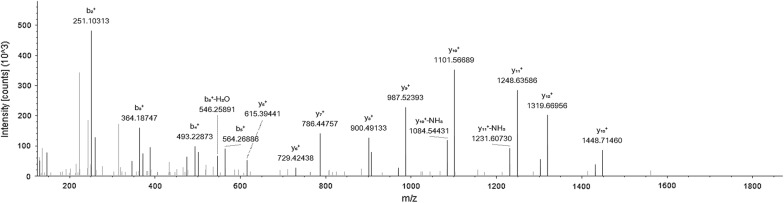
MS2 spectra of the peptide SYLEAFNSNGNNLQLK originating from CUZD1 protein, annotated with b and y ions

**Fig. 4:**
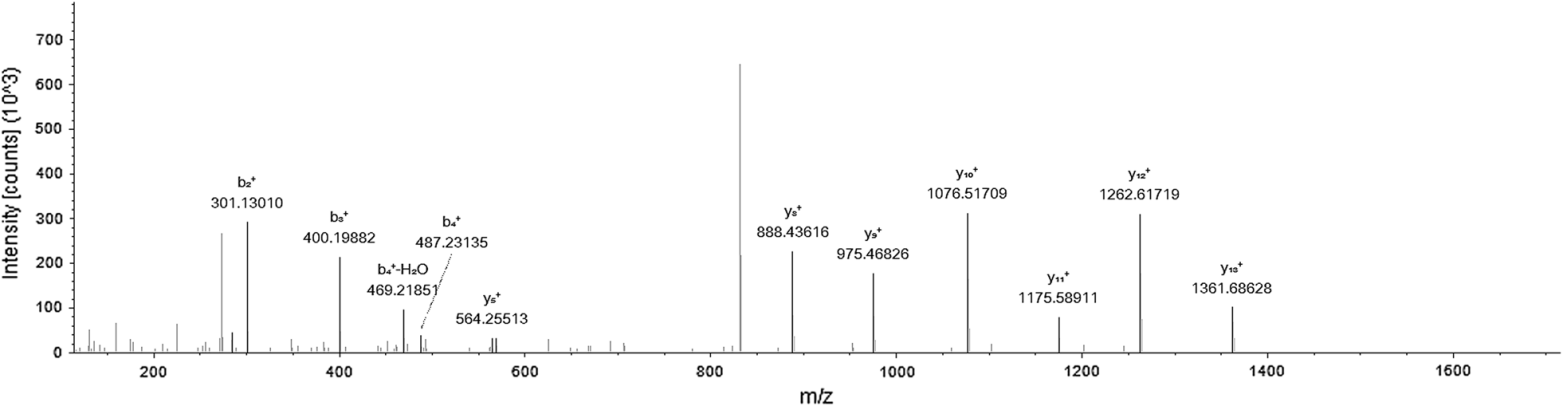
MS2 spectra of peptide NWVSVTSPVQASAcr, originating from GP2 protein, annotated with b and y ions

**Fig. 5:**
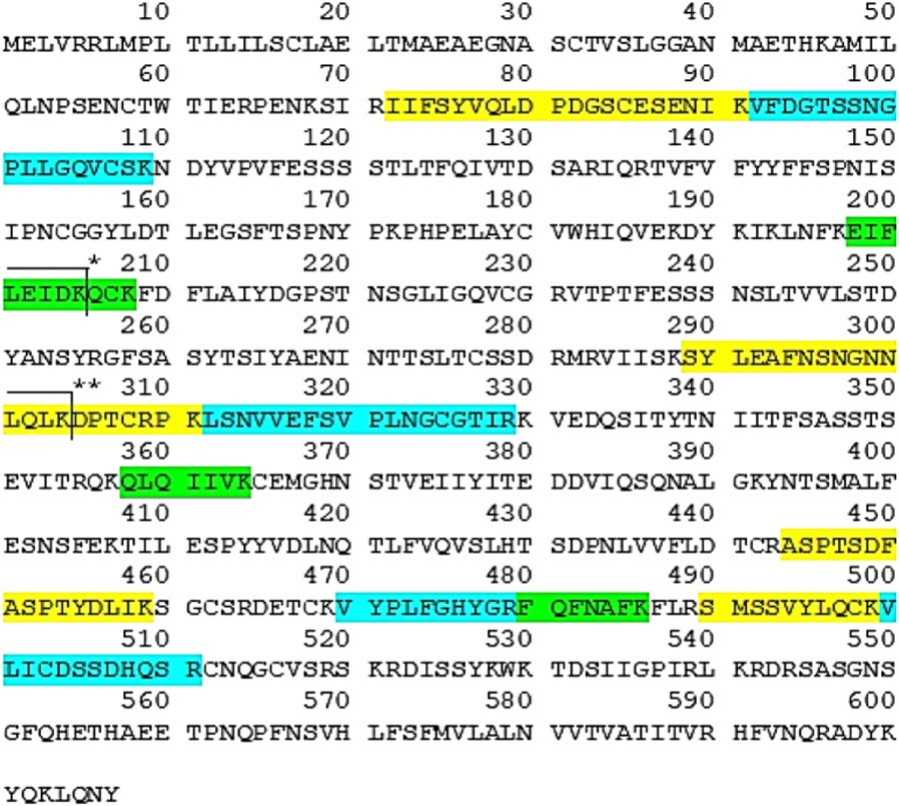
Tryptic peptides corresponding to CUZD1 that were identified by our immuno-MS approach. These peptides are highlighted on the entire CUZD1 protein sequence. *Note: The peptide EIFLEIDK (E198-K205) was also identified as a miscleaved EIFLEIDKQCK (E198-K208). **Note: The peptide SYLEAFNSNGNNLQLK (S289-K304) was also identified as a miscleaved SYLEAFNSNGNNLQLKDPTCRPK (S289-K311). ***Note: The sequence was obtained from UniProt

**Fig. 6:**
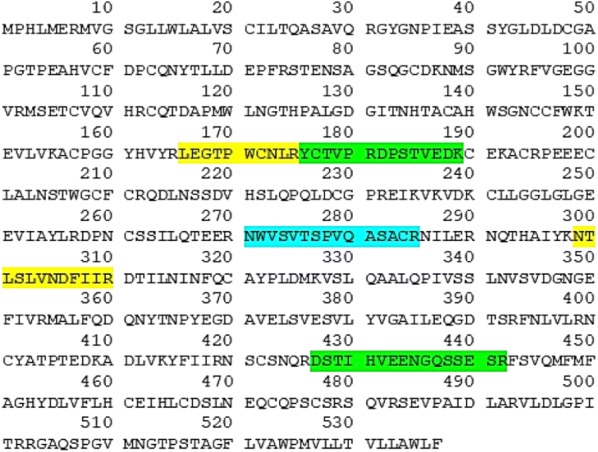
Tryptic peptides corresponding to GP2 that were identified by our immuno-MS approach. These peptides are highlighted on the entire GP2 protein sequence. ***Note: The sequence was obtained from UniProt

We used the protocol of Fig. 1 to immunoaffinity purify proteins on previously captured autoantibodies from serum. Shotgun MS identified CUZD1 in experiments involving serum that was positive for anti-CUZD1 autoantibodies by ELISA. We did not detect CUZD1 as an autoantibody target in negative control serum. 2.5 µl was an adequate volume of anti-CUZD1 autoantibody positive serum to immunoaffinity purify CUZD1 from 100 µg of pancreatic lysate. The number of identified CUZD1 peptides was not dependent on the amount of serum used, if this amount was in the recommended serum volume range (2.5–25 µl). CUZD1 was immunoprecipitated with 25 µl of serum and as little as 20 µg of total protein from pancreatic lysate, but not less. CUZD1 was also immunoprecipitated with 5 µl of positive serum from a 850 µg complex protein lysate containing extracts from 17 different human tissues, including pancreas (Fig. 3). All data shown in Additional files 3, 4, 5, 6, 7, 8, 9, 10, 11, 12, 13, 14, 15, 16: Tables S3–S16. Among other proteins identified by this method, pancreatic secretory granule membrane major glycoprotein 2 (GP2) was also identified in several experiments where CUZD1 was identified (Fig. 4). Across all mentioned experiments (one technical replicate), the identified tryptic peptides for CUZD1 are shown in Fig. 5 and the identified tryptic peptides for GP2 are shown in Fig. 6.

## Discussion

The presented immuno-MS method for identifying protein targets of serum autoantibodies has the potential to become a valuable tool in clinical medicine. This protocol can be effectively used to discover proteome-wide targets of autoantibodies by utilizing complex protein mixtures from human tissues as sources of candidate proteins. The method is based on the ability of mass spectrometry to accurately identify and quantify immunoprecipitated proteins after trypsin digestion. One area of application would be autoimmune diseases for which the autoantibodies involved are unknown. These autoantibodies may serve as diagnostic or prognostic markers or help in disease sub-classification [13]. Autoantibodies could also be used for prediction of therapeutic response or adverse reactions. For example, several clinically relevant autoantibodies have been detected in patients with irAEs following treatment with immune checkpoint inhibitors [14].

One possible limitation of our method is the identification of false positive autoantibody targets or autoantibodies of unknown significance. Our shotgun mass spectrometry analysis yields a candidate list of proteins that were immunoprecipitated on the protein G magnetic beads, but only a fraction are expected to be actual autoantibody targets. The remaining identified proteins likely include proteins that are non-specifically bound to the beads such as highly abundant tissue or serum proteins, as well as frequent protein contaminants with MS such as keratins. Hence, it is necessary to use negative control serum samples to create an exclusion list of non-specific proteins to eliminate from the candidate list. Our methodology can also identify immune complexes that are present in the serum, such as antibodies bound to complement proteins and apolipoproteins [15]. It is mandatory that the initial hits be confirmed using recombinant protein, or with an orthogonal technique such as ELISA. Another limitation is the possibility for false negatives. This may occur if autoantibodies are present in serum but their cognate proteins are not present in the protein mix or present at miniscule amounts. To minimize this risk, we used a protein mixture of lysate from 17 human tissues; we cannot exclude, however, false negative results as the whole human proteome is very likely more diverse. We expect that this method will become even more powerful when equimolar recombinant protein mixes, covering the whole proteome, become available. Some recent efforts are already addressing this issue [16, 17].

In our experiments, the presence of GP2 autoantibodies in several experiments where CUZD1 autoantibodies were also identified is a promising result, since the presence of autoantibodies to GP2 has also been described as a biomarker of CD [11]. GP2 is primarily expressed in the exocrine pancreas, and is overexpressed at the site of a patient’s CD inflammation [18]. This finding further illustrates the reliability of this methodology to effectively immunoaffinity purify and identify autoantibody targets from patient sera.

Immunoproteomic technologies are widely used to discover autoantigens and autoantibodies which may serve as prognostic, diagnostic and theranostic biomarkers [19]. Immunological assays like ELISA are considered the gold standard in clinical settings, and are advantageous due to higher throughput than mass spectrometric analyses [20]. However, MS sensitivity is comparatively high and has the added benefit of not requiring well-characterized and purified antibodies or antigens for assay development. Moreover, MS methods can accurately quantify proteins that may be difficult or even impossible to detect using immunological methods. Some examples include the measurement of protein isoforms in the presence of all isoforms, measuring specific post-translationally modified proteins, and processed forms of proteins in biological samples [20]. Autoantibody testing using methods such as ours is becoming increasingly relevant in this new era of personalized medicine, and should be a continued practice in the search for novel clinical biomarkers.

## Conclusions

This study demonstrates the effectiveness of a proteomic approach to identify serum autoantibody targets, using immunoaffinity purification followed by tandem mass spectrometry. Our protocol was optimized for the immunoaffinity purification of CUZD1 from as little as 20–100 µg of pancreatic lysate using anti-CUZD1 autoantibodies present in 2.5–25 µl of IBD patient sera. GP2, whose autoantibodies are a known biomarker of CD, was also immunoprecipitated from these patient sera, as an additional internal positive control. Our methodology is applicable for proteome-wide analysis of autoantibody targets in a wide variety of clinical settings.

## Additional files

**Additional file 1: Table S1.** Experimental set-up for the immunoprecipitation of CUZD1 using protein G magnetic beads.

**Additional file 2: Table S2.** Concentration of total protein in each human tissue protein lysate, as determined by a Pierce BCA Protein Assay.

**Additional file 3: Table S3.** Mass spectrometry data of experiment with 25 µl of negative serum and 100 µg of pancreatic lysate. Mass spectrometry run 1.

**Additional file 4: Table S4.** Mass spectrometry data of experiment with 25 µl of negative serum and 100 µg of pancreatic lysate. Mass spectrometry run 2.

**Additional file 5: Table S5.** Mass spectrometry data of experiment with 25 µl of positive serum and 100 µg of pancreatic lysate. Mass spectrometry run 1.

**Additional file 6: Table S6.** Mass spectrometry data of experiment with 25 µl of positive serum and 100 µg of pancreatic lysate. Mass spectrometry run 2.

**Additional file 7: Table S7.** Mass spectrometry data of experiment with 25 µl of positive serum and 20 µg of pancreatic lysate. Mass spectrometry run 1.

**Additional file 8: Table S8.** Mass spectrometry data of experiment with 25 µl of positive serum and 20 µg of pancreatic lysate. Mass spectrometry run 2.

**Additional file 9: Table S9.** Mass spectrometry data of experiment with 25 µl of positive serum and 2 µg of pancreatic lysate. Mass spectrometry run 1.

**Additional file 10: Table S10.** Mass spectrometry data of experiment with 25 µl of positive serum and 2 µg of pancreatic lysate. Mass spectrometry run 2.

**Additional file 11: Table S11.** Mass spectrometry data of experiment with 2.5 µl of positive serum and 100 µg of pancreatic lysate. Mass spectrometry run 1.

**Additional file 12: Table S12.** Mass spectrometry data of experiment with 2.5 µl of positive serum and 100 µg of pancreatic lysate. Mass spectrometry run 2.

**Additional file 13: Table S13.** Mass spectrometry data of experiment with 5 µl of positive serum and 50 µg of pancreatic lysate. Mass spectrometry run 1.

**Additional file 14: Table S14.** Mass spectrometry data of experiment with 5 µl of positive serum and 50 µg of pancreatic lysate. Mass spectrometry run 2.

**Additional file 15: Table S15.** Mass spectrometry data of experiment with 5 µl of positive serum and a 850 µg complex tissue lysate. Mass spectrometry run 1.

**Additional file 16: Table S16.** Mass spectrometry data of experiment with 5 µl of positive serum and a 850 µg complex tissue lysate. Mass spectrometry run 2.

## Data Availability

The datasets used and/or analysed during the current study are available from the corresponding author on reasonable request.

## Abbreviations

ABC: ammonium bicarbonate
BSA: bovine serum albumin
CD: Crohn’s disease
CUZD1: CUB, and zona pellucida-like domain-containing protein 1
DTT: dithiothreitol
ELISA: enzyme-linked immunoabsorbent assay
GP2: pancreatic secretory granule membrane major glycoprotein 2
IgG: immunoglobulin G
irAE: immune-related adverse event
immuno-MS: immuno-mass spectrometry
IBD: inflammatory bowel disease
IT: injection time
IAM: iodoacetamide
MAPPing: multiple affinity protein profiling
MS: mass spectrometry
PBS: phosphate buffered saline
SEREX: serological analysis of recombinant tumor cDNA expression libraries
SERPA: serological proteome analysis
