# Correction to: A proteome-wide immuno-mass spectrometric identification of serum autoantibodies

**DOI:** 10.1186/s12014-019-9250-4

**Published:** 2019-07-17

**Authors:** Milena Music, Antoninus Soosaipillai, Ihor Batruch, Ioannis Prassas, Dimitrios P. Bogdanos, Eleftherios P. Diamandis

**Affiliations:** 1 Department of Laboratory Medicine and Pathobiology, University of Toronto, Toronto, Canada; 2 Department of Pathology and Laboratory Medicine, Mount Sinai Hospital, 60 Murray St [Box 32]; Flr 6 - Rm L6-201-1, Toronto, ON M5T 3L9 Canada; 3 Department of Rheumatology and Clinical Immunology, Faculty of Medicine, School of Health Sciences, University of Thessaly, Biopolis, 41110 Larissa, Greece; 4 Department of Clinical Biochemistry, University Health Network, Toronto, Canada; 5 Lunenfeld-Tanenbaum Research Institute, Mount Sinai Hospital, 60 Murray St [Box 32]; Flr 6 - Rm L6-201-1, Toronto, ON M5T 3L9 Canada

## Correction to: Clin Proteom (2019) 16:25 10.1186/s12014-019-9246-0

In the original version of the article [1], an error was noticed under the heading “Immunoprecipitation on protein G magnetic beads” in “Methods” section. The unit symbol in the text that reads as “> 27 µl human” should read as “> 27 µg human”.
