# Supplementary material for: A proteome-wide immuno-mass spectrometric identification of serum autoantibodies
Source: Clin Proteomics. 2019 Jun 20;16:25. doi: 10.1186/s12014-019-9246-0 (PMC6585069; doi:10.1186/s12014-019-9246-0)
Supplement: Supplementary file 1 — Additional file 1: Table S1. Experimental set-up for the immunoprecipitation of CUZD1 using protein G magnetic beads. [file 12014_2019_9246_MOESM1_ESM.docx]

**Supplementary Table 1**. Experimental set-up for the immunoprecipitation of CUZD1 on protein G magnetic beads from tissue lysate using serum with or without anti-CUZD1 autoantibodies.

| Experiment # | Serum Volume | Protein Amount from Tissue Lysate | Protein G Magnetic Beads Slurry Volume |
| --- | --- | --- | --- |
| 1 | 5 µl of positive anti-CUZD1 autoantibody serum | 50 µg from pancreatic lysate | 200 µl |
| 2 | 5 µl of positive anti-CUZD1 autoantibody serum | 850 µg from 17 human tissue lysate mixture (50 µg from each tissue) | 200 µl |
| 3 | 25 µl of positive anti-CUZD1 autoantibody serum | 100 µg from pancreatic lysate | 100 µl |
| 4 | 25 µl of negative anti-CUZD1 autoantibody serum | 100 µg from pancreatic lysate | 100 µl |
| 5 | 2.5 µl of positive anti-CUZD1 autoantibody serum | 100 µg from pancreatic lysate | 100 µl |
| 6 | 25 µl of positive anti-CUZD1 autoantibody serum | 20 µg from pancreatic lysate | 100 µl |
| 7 | 25 µl of positive anti-CUZD1 autoantibody serum | 2 µg from pancreatic lysate | 100 µl |
