# Supplementary material for: A proteome-wide immuno-mass spectrometric identification of serum autoantibodies
Source: Clin Proteomics. 2019 Jun 20;16:25. doi: 10.1186/s12014-019-9246-0 (PMC6585069; doi:10.1186/s12014-019-9246-0)
Supplement: Supplementary file 2 — Additional file 2: Table S2. Concentration of total protein in each human tissue protein lysate, as determined by a Pierce BCA Protein Assay. [file 12014_2019_9246_MOESM2_ESM.docx]

**Supplementary Table 2.** Concentration of total protein in each human tissue protein lysate, as determined by a Pierce BCA Protein Assay.

| **Tissue** | **Concentration of total protein in extract (mg/ml)** |
| --- | --- |
| Pancreas | 13.0 |
| Lung | 4.2 |
| Esophagus | 1.4 |
| Gallbladder | 3.7 |
| Heart | 3.5 |
| Adipose tissue | 1.1 |
| Small intestine | 2.7 |
| Skeletal muscle | 2.3 |
| Endometrium | 10.5 |
| Prostate | 5.7 |
| Breast | 5.7 |
| Spleen | 7.8 |
| Bone marrow | 2.6 |
| Bladder | 3.3 |
| Duodenum | 2.7 |
| Stomach | 1.9 |
| Kidney | 8.2 |
